# CD109 is associated with an immunosuppressive microenvironment and M2 macrophage polarization: pan-cancer analysis and functional validation

**DOI:** 10.1186/s12885-026-16100-4

**Published:** 2026-05-01

**Authors:** Fangqiong Li, Wei Zhang, Xiaoting Gong, Dan Liu, Qi You, Ying Li, Guizhi Zhao, Wei Wang

**Affiliations:** 1 Department of Clinical Laboratory, Tongde Hospital of Zhejiang Province, Zhejiang Chinese Medical University, No.234, Gucui Road, Hangzhou, Zhejiang 310012 China; 2 Department of Pharmacy, Tongde Hospital of Zhejiang Province Affiliated to Zhejiang Chinese Medical University, Hangzhou, 310012 China; 3 Department of Science and Education, Tongde Hospital of Zhejiang Province, Zhejiang Chinese Medical University, Hangzhou, Zhejiang 310012 China

**Keywords:** CD109, Tumor-immune microenvironment, Pan-cancer analysis, Macrophage polarization, Prognostic heterogeneity, Immune suppression

## Abstract

**Background:**

CD109 is a glycosylphosphatidylinositol‑anchored glycoprotein implicated in tumor progression and physiological homeostasis. Although aberrant CD109 expression has been reported in multiple malignancies, its prognostic relevance across cancer types and its potential immunomodulatory roles remain incompletely characterized.

**Methods:**

We performed an integrative pan-cancer analysis of CD109 using RNA sequencing data from the Genotype-Tissue Expression (GTEx) and The Cancer Genome Atlas (TCGA) databases. CD109 expression, genetic alterations, survival associations, and immune infiltration patterns were systematically assessed. Single-cell RNA sequencing (scRNA-seq) data from non-small cell lung cancer (NSCLC) cohorts were analyzed to define cellular CD109 distribution. Functional validation was performed using siRNA-mediated CD109 knockdown in A549 lung adenocarcinoma cells, followed by macrophage co-culture experiments, flow cytometry, qPCR, ELISA, and migration assays.

**Results:**

CD109 was markedly upregulated in 15 tumor types and showed heterogeneous prognostic associations across cancers. Elevated CD109 expression was associated predominantly with unfavorable survival in several tumor types, whereas opposite prognostic associations were observed in a subset of cancers. Bioinformatic analysis revealed that high CD109 expression was associated with an immunosuppressive tumor immune microenvironment, characterized by the enrichment of M2-like tumor-associated macrophages (TAMs) and activation of oncogenic signaling axes. Single-cell profiling showed that CD109 was predominantly expressed in malignant cells and a subset of M2 macrophages. Consistent with these clinical and computational insights, functional validation in an in vitro lung cancer model showed that CD109 knockdown in A549 cells significantly suppressed tumor migration and was associated with a shift in macrophage polarization away from a pro-tumorigenic M2 phenotype toward an anti-tumor M1 state.

**Conclusions:**

Our study highlights CD109 as a context-dependent biomarker with tumor type specific prognostic relevance and links its expression to immunosuppressive features of the tumor immune microenvironment. These findings suggest that CD109 warrants further investigation as a potential therapeutic target, particularly in tumor contexts characterized by macrophage-associated immunosuppression, although the macrophage-related functional effects observed here were validated in a lung cancer model and require further investigation in additional tumor types.

**Supplementary Information:**

The online version contains supplementary material available at 10.1186/s12885-026-16100-4.

## Introduction

The tumor immune microenvironment (TIME) has emerged as a key factor influencing cancer initiation, progression, and metastasis, as well as response to therapeutic interventions [1–3]. This dynamic ecosystem is composed of various immune cell populations together with soluble factors such as cytokines, chemokines, and growth factors, which regulate the interplay between antitumor activity and immune evasion [4–6]. An immunosuppressive TIME is characterized by the expansion of M2 tumor associated macrophages (TAMs) and other inhibitory immune populations that foster tumor growth, angiogenesis, and suppress adaptive immunity [7–11]. Recent studies in lung adenocarcinoma have further shown that tumor-intrinsic molecular alterations can shape distinct TIME states, with immunosuppressive subtypes often characterized by myeloid-cell enrichment, including increased macrophage infiltration, and associated with unfavorable clinical outcomes [12, 13]. Despite advances in immune checkpoint blockade, sustained immunosuppressive signaling continues to hinder therapeutic efficacy, particularly in NSCLC and other recalcitrant malignancies [14–16]. Identifying molecular drivers that shape this suppressive landscape is therefore imperative.

CD109, a glycosylphosphatidylinositol-anchored cell surface protein, has emerged as a multifaceted player in oncology [17, 18]. It was initially identified as being expressed on blood cells, including activated T cells, platelets, and a subset of CD34⁺ bone marrow cells [19]. CD109 is aberrantly expressed in cancers such as lung cancer [20], glioma [21], and squamous cell carcinoma [22]. Functionally, CD109 interacts with multiple oncogenic signaling axes, including TGF‑β, IL‑6/STAT3, JAK‑STAT3, and Hippo pathways, thereby promoting epithelial‑mesenchymal transition (EMT), acquisition of stemness, enhanced protease activity, and immune escape [21–24]. Many of these processes converge on mechanisms underlying TIME suppression, suggesting that CD109 may be associated with immune-contextural remodeling [22]. Clinically, elevated CD109 expression correlates with poor prognosis in squamous and breast carcinomas [25–27], highlighting its translational potential as a biomarker of tumor aggressiveness. Our previous work demonstrated that CD109 is highly expressed in NSCLC cells and can be downregulated by triptolide, a bioactive compound derived from traditional Chinese medicine, confirming its pharmacological tractability [28]. Nevertheless, the specific role of CD109 in modulating the tumor immune microenvironment, particularly its impact on macrophage polarization, has not been comprehensively elucidated. Although emerging studies suggest a link between CD109 and immune suppression [29, 30], systematic analyses across cancer types and cellular contexts are still lacking.

Given its involvement in multiple oncogenic pathways that intersect with immune regulation, we hypothesized that CD109 may be associated with the formation of an immunosuppressive TIME, partly through a potential role in M2‑like macrophage polarization. We performed an integrative multi‑omics investigation combining pan‑cancer transcriptomic analyses, immune‑correlation modeling, single‑cell profiling, and in vitro co‑culture validation. We first performed pan-cancer analysis of CD109 using data from TCGA and the GTEx project to delineate its expression landscape, clinical relevance, and prognostic significance across 33 cancer types. We then employed bioinformatics approaches, including gene set enrichment analysis (GSEA) and multiple immune deconvolution algorithms implemented in TIMER2 and ImmuCellAI, to characterize CD109 associated biological pathways and assess its correlation with immune cell infiltration and tumor immune microenvironment features, with a special focus on TAMs. Furthermore, we leveraged scRNA-seq data from NSCLC cohorts to define the cellular sources and expression dynamics of CD109 within the complex cellular tapestry of the TME. Finally, we conducted in vitro co-culture experiments using a transwell system with A549 NSCLC cells and THP-1-derived macrophages to investigate the role of tumor cell-intrinsic CD109 in modulating macrophage polarization and its potential association with tumor cell migration.

The present study sought to comprehensively elucidate CD109, with a focus on its immunomodulatory role. We anticipate that our findings will offer valuable insights into the potential of CD109 as a biomarker and as a candidate target for future immunotherapy‑oriented studies, while the mechanistic observations reported here should be interpreted within the NSCLC model used for experimental validation.

## Materials and methods

### Data sources

UCSC Xena platform was used to obtain mRNA expression data and corresponding clinical information from TCGA and the GTEx project, covering 33 cancer types and normal tissues. TCGA data were primarily used for tumor samples and available adjacent normal tissues, whereas GTEx data were incorporated as an additional source of normal tissue controls for tumor types with limited or no normal samples in TCGA. CD109 genetic alteration profiles were collected from cBioPortal (http://www.cbioportal.org). Tumor cell expression profiles and drug half‑maximal inhibitory concentration (IC50) data were obtained from the Genomics of Drug Sensitivity in Cancer database (www.cancerRxgene.org). For single‑cell analyses, we downloaded scRNA‑seq datasets from TISCH (tisch.comp-genomics.org/), including NSCLC_GSE99254 and NSCLC_GSE127465.

### Analysis of TCGA pan-cancer data

We performed an integrated pan-cancer analysis based on TCGA data to systematically characterize the expression pattern, prognostic significance, and potential biological functions of CD109 across human cancers. For expression analysis, TCGA primary tumor tissues were compared with TCGA adjacent normal tissues when available, and GTEx normal tissues were included as supplementary controls for cancer types lacking sufficient TCGA normal samples. Batch effects between TCGA and GTEx were corrected using the ComBat algorithm in the “sva” R package. Sample numbers for each cancer type are listed in Supplementary Table 1. To validate the integrated analysis, paired tumor and adjacent normal tissues from TCGA were further analyzed in cancer types with available matched samples (Supplementary Fig. 1).

Uni-variate survival analyses were used in the R packages “survival” and “survminer” to assess associations between CD109 expression and overall survival (OS), disease-specific survival (DSS), disease-free interval (DFI), and progression-free interval (PFI). To further evaluate whether CD109 had independent prognostic value, multivariable Cox proportional hazards regression analyses were performed in selected representative tumor types with available clinical annotation. Response groups were defined according to the original annotations, and patients were further stratified into CD109-high and CD109-low groups based on the median expression level of CD109 in each cohort. Survival differences were evaluated by Kaplan-Meier analysis with the log-rank test, and CD109 expression was compared between response groups. Functional enrichment analyses were performed using the “GSVA” and “clusterProfiler” R packages based on gene sets from the MSigDB Hallmark collection, KEGG, GO, and Reactome databases. For drug-response analysis, CD109 expression profiles in cancer cell lines were integrated with GDSC IC50 data, and correlations between CD109 expression and drug IC50 values were calculated using Spearman’s rank correlation. Significant associations were summarized by representative drug classes and interpreted as reduced drug sensitivity rather than direct evidence of generalized resistance.

### TME analysis

Multiple computer algorithms were used to examine the tumor’s immunological and stromal components. The ESTIMATE R program was used to compute the immune score, stromal score, and tumor purity for each tumor sample using transcriptome data. Immune deconvolution results were gathered from the TIMER2 (http://timer.cistrome.org/) and ImmuCellAI databases, as well as previously published studies. The ImmuCellAI platform evaluated the relative abundance of tumor-infiltrating immune cell subsets using the single-sample Gene Set Enrichment Analysis (ssGSEA) approach. TIMER2 used six established computational algorithms (TIMER, CIBERSORT, MCP-counter, quanTIseq, xCell, and EPIC) to determine immune infiltration levels. The TIMER2 platform standardized the results.

### scRNA-seq analysis

ScRNA-seq data from two independent NSCLC cohorts (GSE99254 and GSE127465) were obtained from the TISCH database and analyzed using Seurat (R package). Batch effects between datasets were corrected using Harmony (R package). Following normalization with ScaleData, principal component analysis (PCA) was performed, and dimensionality reduction was visualized using UMAP. Cell clustering was performed at a resolution of 0.8, and differentially expressed genes for each cluster were identified using FindAllMarkers. Cell types were annotated by comparison to the TISCH database annotations.

To assess pathway activity differences, HALLMARK gene sets were downloaded from MSigDB (http://www.gsea-msigdb.org/gsea/index.jsp), and pathway scores were calculated using GSVA (R package). Cell-cell communication analysis was performed using CellChat (R package). To investigate the association between CD109 expression and TME composition, average CD109 expression across all cells within each sample was calculated, and samples were classified into CD109-high and CD109-low groups according to the median value. This stratification was used to compare differences in cellular composition and intercellular communication patterns between samples with relatively high versus low overall CD109 expression, rather than to infer a cell-intrinsic effect of CD109. Cell-type proportions were then compared between groups.

### Cell culture and gene knockdown

Human lung cancer A549 cells (ATCC^®^CCL‑185™) and human monocytic leukemia THP‑1 cells (MeiXuan Biological Science Co., Ltd., Shanghai, China) were maintained in RPMI‑1640 medium (Hyclone, USA) supplemented with 10% fetal bovine serum (FBS; TransGen, China), 100 U/mL penicillin, and 100 U/mL streptomycin (Gibco, USA) at 37 °C in a humidified incubator with 5% CO₂. All cell lines were authenticated by short tandem repeat (STR) profiling and confirmed to be free of mycoplasma contamination.

A549 cells were transfected with three independent CD109-targeting siRNAs or a non-targeting negative control siRNA (siNC) (Shanghai Sangon Biotech, China; Supplementary Table 2) using Lipofectamine 2000 (Thermo Fisher Scientific, USA) according to the manufacturer’s protocol. Cells were seeded in six-well plates (4 × 10^5^ cells/mL) one day before transfection and transfected at 80–95% confluence in antibiotic-free medium. For each well, 10 µl Lipofectamine 2000 and 5 µl siRNA were separately diluted in serum-free medium, combined, and incubated for 20 min at room temperature before being added dropwise to cells. After 5–6 h, the medium was replaced with complete medium, and knockdown efficiency was assessed 48–72 h post-transfection by qPCR and Western blot.

Total RNA was extracted using the RNeasy Mini Kit (Qiagen, Germany), reverse‑transcribed using a cDNA synthesis kit (CWBio, China), and analyzed by qPCR with gene‑specific primers (Supplementary Table 3). Relative gene expression levels were calculated using the 2⁻^ΔΔCt^. Total protein was extracted using RIPA buffer (Beyotime, China), quantified by BCA assay (Beyotime, China), and separated by 12% sodium dodecyl sulfate‑polyacrylamide gel electrophoresis (SDS‑PAGE, 20 µg per lane), followed by transfer to polyvinylidene fluoride (PVDF) membranes. Membranes were incubated overnight at 4 °C with primary antibodies against CD109 (1:1000, Proteintech, USA) and GAPDH (1:2000, Cell Signaling Technology, USA), followed by appropriate secondary antibodies. Experiments were performed with three independent biological replicates.

### Differentiation and generation of macrophages

M0 macrophages were generated by treating THP‑1 cells with 100 nM phorbol 12‑myristate 13‑acetate (PMA; Solarbio, China) for 24 h. To induce TAMs, a 0.4‑µm pore size Transwell co‑culture system (Corning, USA) was used. Successful differentiation was confirmed by morphological changes and upregulation of CD11b and CD68 expression.PMA‑differentiated macrophages were co‑cultured with A549 cells from different groups (NC‑A549, siNC‑A549, and siCD109‑A549). After 24 h of co‑culture, macrophages were collected for subsequent TAM‑related analyses.

### Flow cytometry

Macrophages were harvested from six‑well plates, washed, and resuspended as single‑cell suspensions. Cells were incubated for 30 min at 4 °C with fluorochrome‑conjugated antibodies against CD11b‑FITC, CD68‑APC, CD86‑PE, and CD206‑PE/Cy7 (BioLegend, USA). After two washes, cells were resuspended in 200 µL of flow cytometry buffer and analyzed on an EXflow206 flow cytometer (DAKEWE, China). Data were processed and analyzed using FlowJo software (FlowJo, USA).

### Enzyme‑linked immunosorbent assay (ELISA) analysis

After 24 h of co‑culture, culture supernatants from NC‑A549, siNC‑A549, and siCD109‑A549 groups were collected and centrifuged at 3000 rpm for 10 min to remove cellular debris. Concentrations of TGF‑β, IL‑10, and IL‑12 were measured using commercial human ELISA kits (Shanghai Meixuan, China) according to the manufacturers’ instructions. All experiments were performed in triplicate.

### CCK-8 assay

A549 cells in the NC, siNC, and siCD109 groups were seeded into 96-well plates at an appropriate density after transfection. The 24-h time point was selected to match the duration of the Transwell migration assay. Cell viability was evaluated using a Cell Counting Kit-8 (Beyotime, China) according to the manufacturer’s protocol. All experiments were performed in triplicate.

### Transwell analysis

Transwell migration assays were performed using 24‑well Transwell inserts. The upper chambers were seeded with A549 cells (5 × 10⁵ cells/well) from the following groups: NC‑A549, siNC‑A549, siCD109‑A549 (co‑culture), and siCD109‑A549 (monoculture). For co‑culture groups, M0 macrophages were added to the lower chambers, whereas only culture medium was added to the lower chambers of the monoculture group. After 24 h of incubation, migrated cells were fixed with 4% paraformaldehyde (Dingguo, China) at room temperature for 20 min and stained with crystal violet solution (Solarbio, China). Cells in five randomly selected fields were counted under a microscope.

### Statistical analysis

Bulk RNA-seq data were analyzed using non-parametric approaches. Two-group differences were assessed with the Mann-Whitney U test, while comparisons across multiple groups employed the Kruskal-Wallis test followed by Dunn’s correction. Spearman’s rank correlation quantified relationships between CD109 levels and immune-related features. Survival outcomes were evaluated by dividing patients into high- and low-CD109 subgroups according to the median expression value calculated separately for each cancer type. Kaplan-Meier survival curves were generated and compared via log-rank tests for OS, DSS, DFI, and PFI. Univariate Cox regression provided HRs with 95% CIs. To address multiple comparisons in gene set and pathway enrichment studies, we applied Benjamini-Hochberg FDR adjustment (q < 0.05). All computational work was conducted in R version 4.5.0, utilizing packages including survival, survminer, Seurat, Harmony, GSVA, clusterProfiler, and CellChat.

For in vitro experiments, differences between groups were analyzed using one-way ANOVA with Dunnett’s post hoc test or two-way ANOVA with Sidak’s post hoc test, as appropriate. Non-normally distributed data were analyzed using the Kruskal-Wallis test with Dunn’s correction. Data are mean ± SD from ≥ 3 replicates; A *P-value < 0.05* was considered statistically significant. GraphPad Prism 10 performed analyses.

## Results

### CD109 is dysregulated and associated with genetic and epigenetic alterations across cancers

**Fig. 1:**
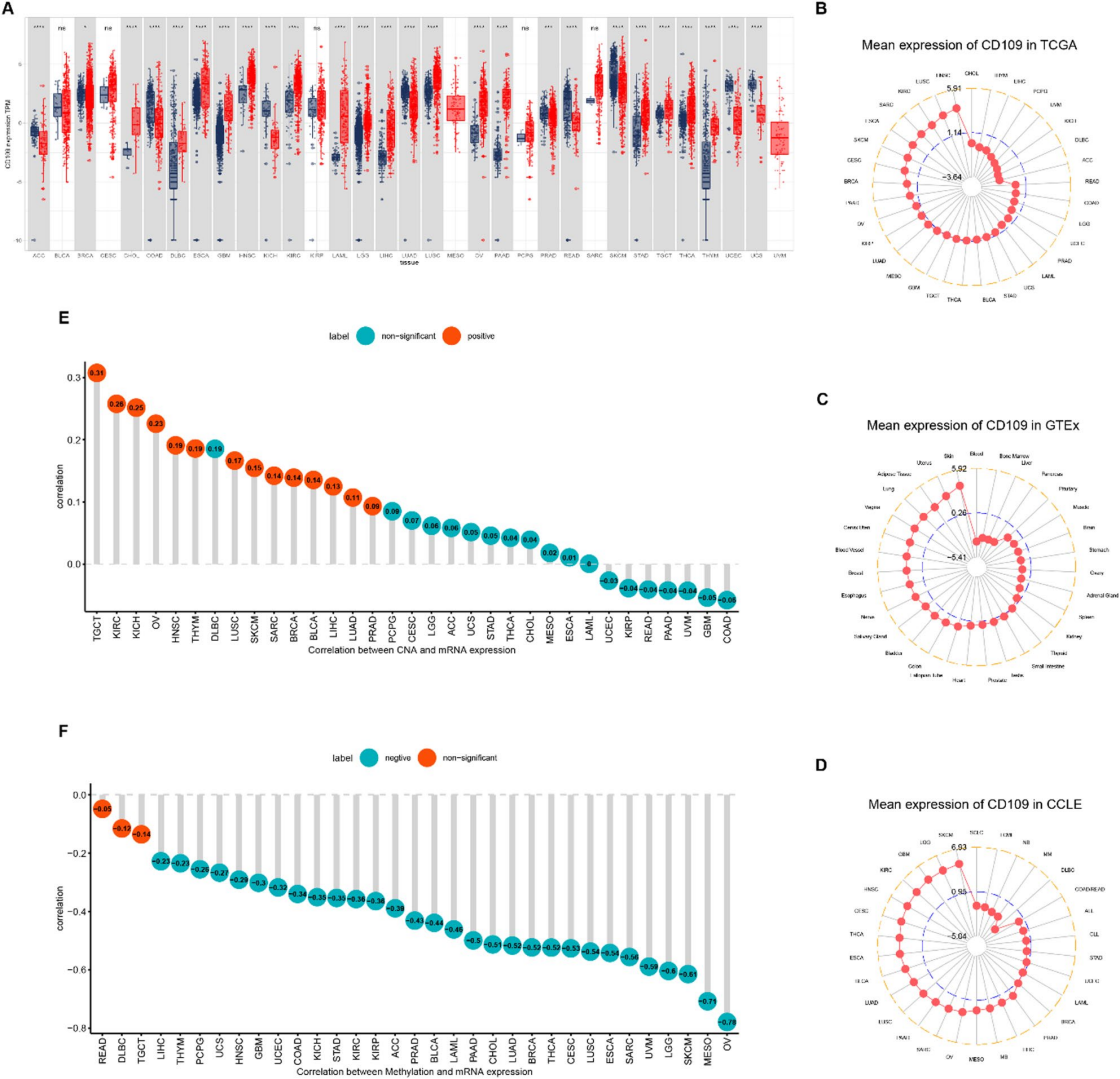
CD109 expression and genomic/epigenetic regulation across cancers. **a** Pan-cancer expression of CD109. **b**–**c** CD109 expression in TCGA, GTEx, and CCLE datasets. **d** CD109 expression in tumor cell lines from the CCLE cohort. **e** Correlation between CD109 and copy number amplification. **f** Correlation between CD109 and DNA methylation levels. *P  < 0.05, **P  < 0.01, ***P  < 0.001, ****P  < 0.0001

CD109 mRNA expression was assessed across TCGA, GTEx and CCLE datasets. Integrated TCGA-GTEx analysis showed that CD109 was significantly upregulated in multiple tumor types relative to normal tissues (Fig. 1a-c). This pattern was further validated in paired tumor and adjacent normal tissues from the TCGA cohort, which also showed higher CD109 expression in tumors (Supplementary Fig. 1). CD109 expression also exhibited substantial heterogeneity across cancer cell lines (Fig. 1d). Correlation analysis revealed a positive association between CD109 expression and copy number amplification and a negative association with DNA methylation levels (Fig. 1e–f), suggesting that CD109 dysregulation is associated with both genetic and epigenetic alterations across cancers. Together, these results identify CD109 as a broadly dysregulated gene across human malignancies.

### Prognostic significance and CD109 associated signaling pathways across cancers

**Fig. 2:**
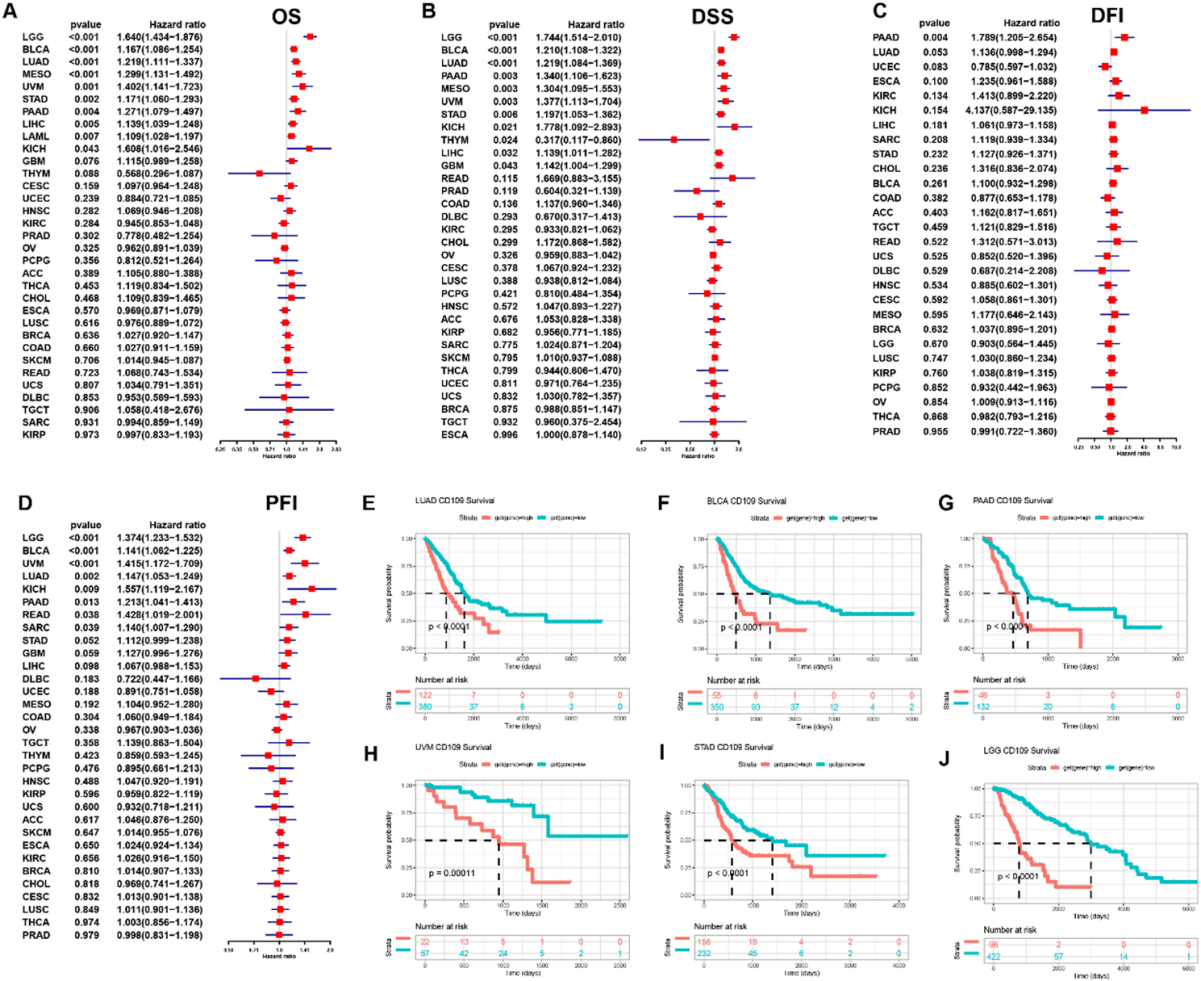
Prognostic analyses of CD109 expression. **a**–**d** Univariate Cox analyses for overall survival (OS), disease-specific survival (DSS), disease-free interval (DFI), and progression-free interval (PFI) across cancers. **e**–**j** Kaplan–Meier curves in representative tumor types. Complete survival curves for all 33 cancer types are provided in Supplementary Fig. 2. *P  < 0.05, **P  < 0.01, ***P  < 0.001, ****P  < 0.0001

Univariate Cox regression analyses across TCGA tumor types showed that elevated CD109 expression was significantly associated with worse overall survival (OS), disease‑specific survival (DSS), disease‑free interval (DFI), and progression‑free interval (PFI) in multiple cancers (Fig. 2a–d). Kaplan-Meier curves further supported an unfavorable association between high CD109 expression and OS in 12 cancer types, whereas inverse associations were observed in 7 additional tumors (Fig. 2e–j; Supplementary Fig. 2). Overall, these univariate survival analyses indicate that CD109 exhibits heterogeneous and tumor-type-specific prognostic relevance across cancers.To further determine whether CD109 retains independent prognostic value after adjustment for established clinical confounders, multivariable Cox regression analyses were performed in five representative cancer types with relatively complete clinical annotations. After adjustment for age and stage-related variables, CD109 remained an independent adverse prognostic factor in BLCA, LUAD, and PAAD. By contrast, CD109 was not independently associated with OS in KIRC or UCEC after multivariable adjustment (Table 1). Together, these findings suggest that the prognostic impact of CD109 is cancer-type specific and remains independent only in a subset of tumors.

**Table 1:** Multivariable Cox regression analyses of CD109 in five representative cancer types

Cancer type	Covariates	HR (95% CI)	*P* value	C-index (95% CI)
BLCA	Age, pT stage, pN stage, pM stage	1.307(1.133–1.508)	2.47E-04	0.691(0.645–0.737)
LUAD	Age, pT stage, pN stage, pM stage	1.328(1.132–1.557)	5.06E-04	0.685 (0.636–0.733)
PAAD	Age, pT stage, pN stage, pM stage	1.323(1.082–1.618)	6.43E-03	0.629 (0.566–0.693)
KIRC	Age, pT stage, pN stage, pM stage	0.940(0.742–1.190)	6.07E-01	0.775 (0.729–0.820)
UCEC	Age, race, grade	0.948(0.750–1.198)	6.56E-01	0.682 (0.628–0.736)

**Fig. 3:**
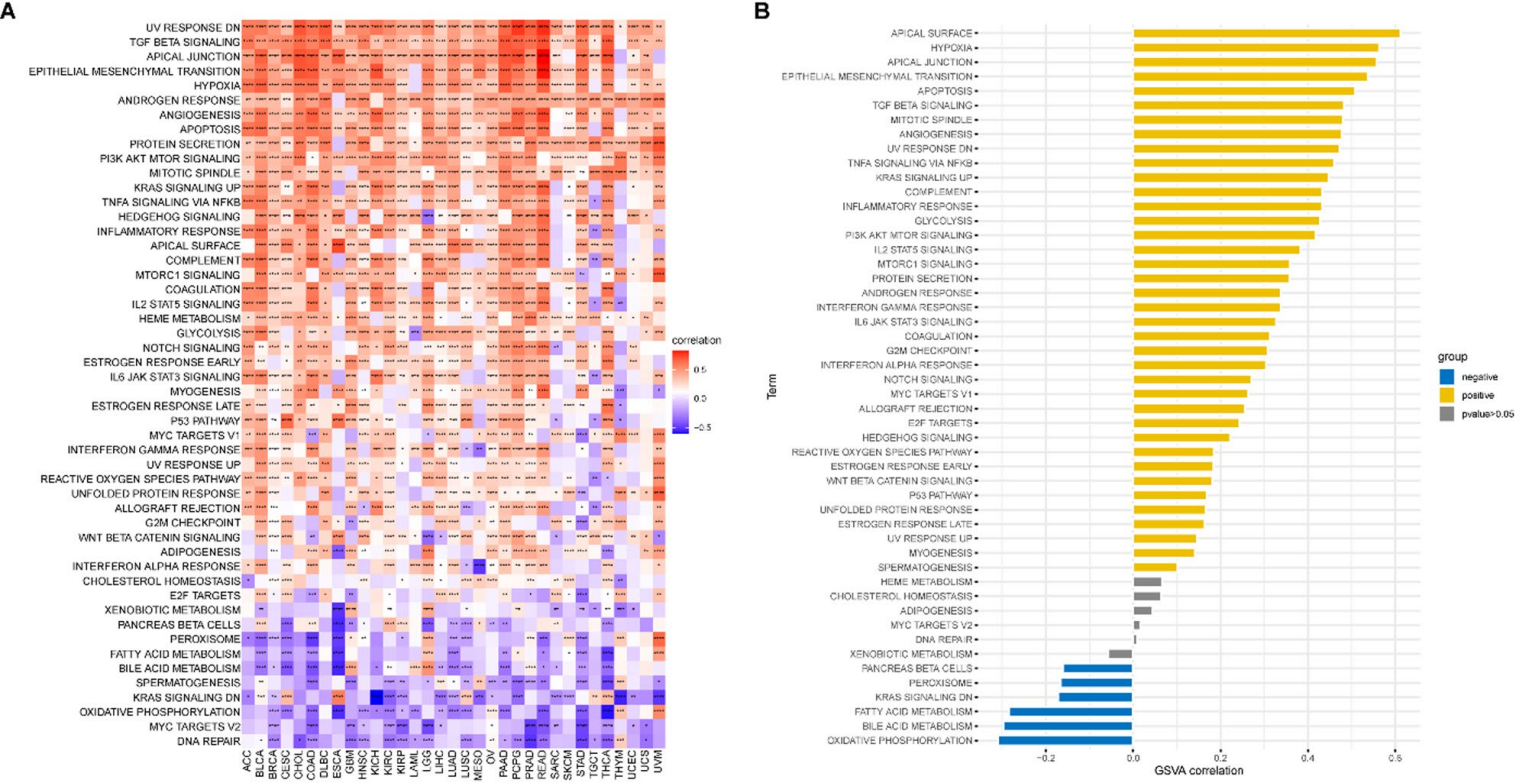
Functional enrichment of CD109-associated pathways. **a** GSVA heatmap showing enrichment scores for 50 Hallmark pathways in pan-cancer datasets. **b** Representative LUAD GSVA plot illustrating activation of TGF-β, EMT, and hypoxia signaling related to CD109

To explore the biological pathways associated with these prognostic patterns, Gene Set Variation Analysis (GSVA) was performed using Hallmark pathways in pan-cancer and LUAD datasets. High CD109 expression was positively associated with TGF-β signaling, EMT, hypoxia, and angiogenesis (Fig. 3a-b). Complementary GSEA, GO, and KEGG/Reactome analyses further revealed enrichment of processes related to extracellular matrix organization, ECM receptor interaction, focal adhesion, PI3K-Akt signaling, collagen formation, and degradation (Supplementary Fig. 3). Collectively, these results suggest that CD109 is linked to key oncogenic and immunoregulatory pathways involved in tumor progression and microenvironment remodeling, particularly those related to cell-ECM interaction and TGF-β-associated signaling.

### Association of CD109 expression with immunosuppressive TME features and immune‑cell infiltration

**Fig. 4:**
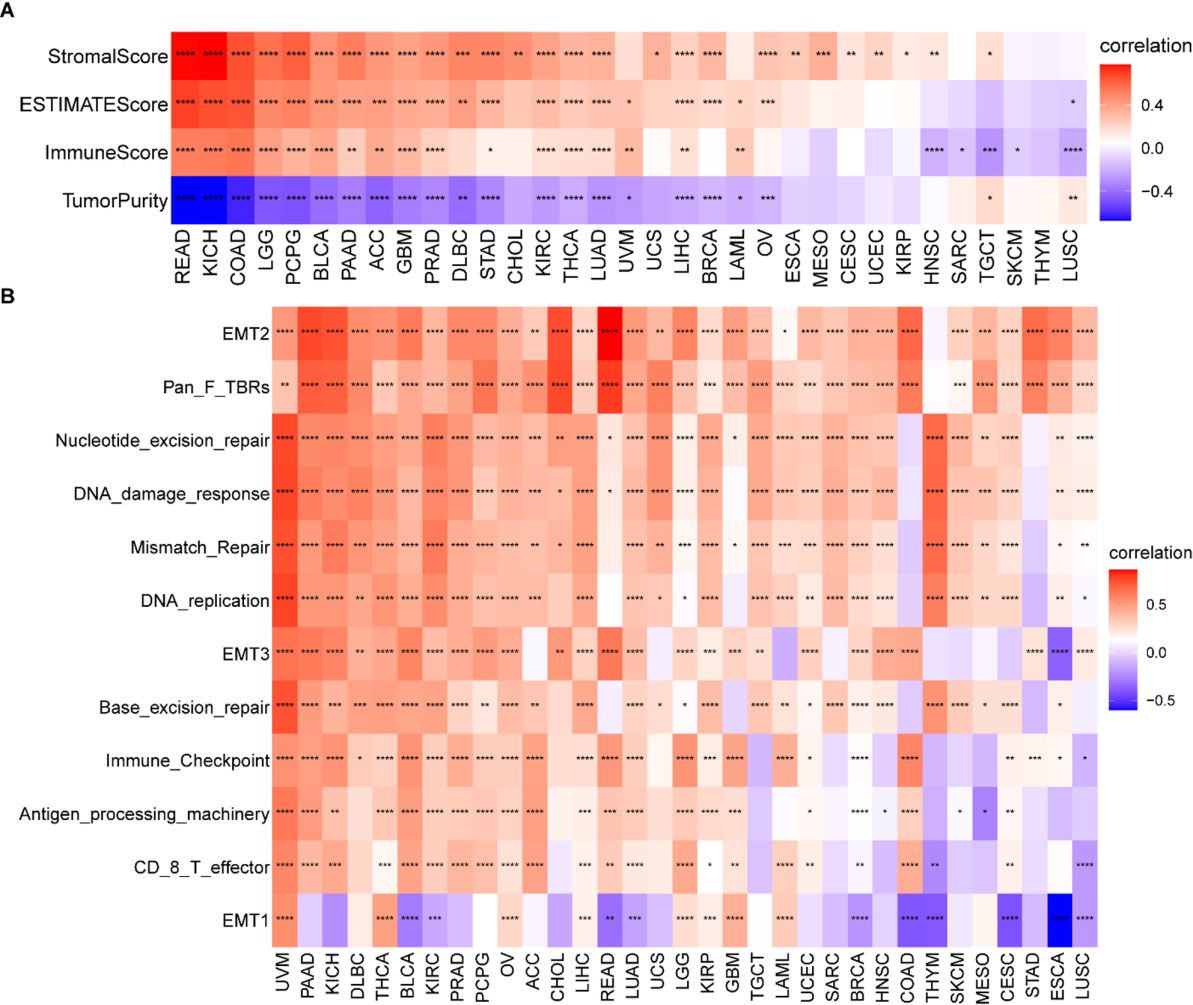
Association of CD109 with TME features. **a** Scatterplots showing correlations between CD109 expression and ESTIMATE scores (immune, stromal, and purity). **b** Correlations between CD109 and markers of macrophages and fibroblasts. *P  < 0.05, **P  < 0.01, ***P  < 0.001, ****P  < 0.0001

Across tumors, CD109 expression correlated positively with immune and stromal scores and negatively with tumor purity (Fig. 4a). It also showed consistent associations with immune and cell-cycle-related signatures (Fig. 4b), suggesting that CD109-high tumors possess enriched stromal and immune components.

**Fig. 5:**
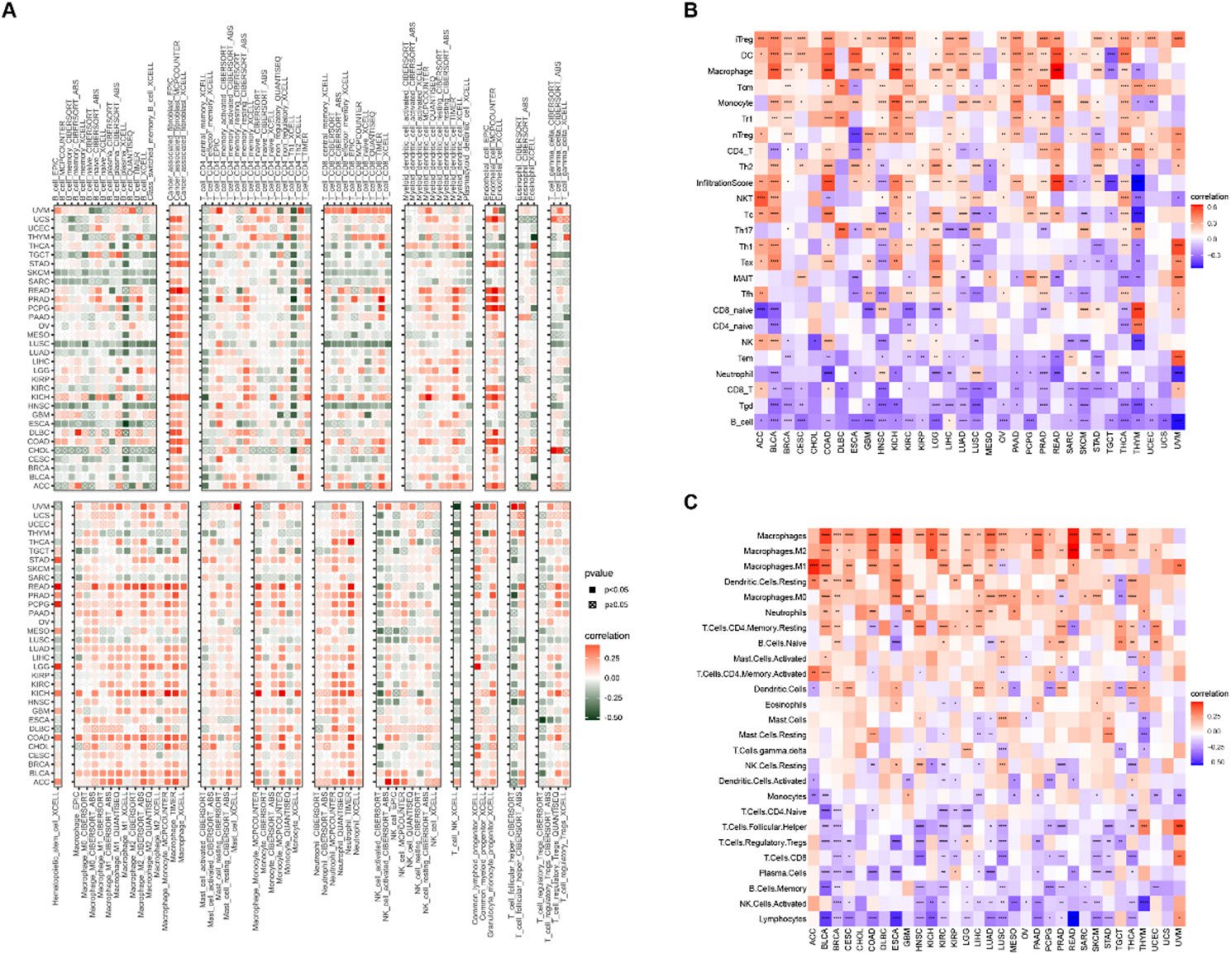
Immune cell infiltration associated with CD109 expression. **a**–**c** Correlation between CD109 and immune cell abundances derived from TIMER2 and ImmuCellAI datasets, indicating higher M2 macrophage and Treg infiltration and reduced CD8⁺ T-cell density. *P  < 0.05, **P  < 0.01, ***P  < 0.001

Immune-cell infiltration analyses using TIMER2 and ImmuCellAI revealed that CD109 was positively associated with tumor-associated macrophages (TAMs), cancer-associated fibroblasts (CAFs), and induced regulatory T cells (iTregs), while showing only weak correlations with CD8⁺ cytotoxic T cells (Fig. 5a-c). Analyses of independent cohorts confirmed the association with increased TAM abundance.

Furthermore, across cancer types, CD109 expression correlated with immunosuppressive-gene, chemokine, and chemokine-receptor signatures (Supplementary Fig. 4a–c) as well as stemness indices (mDNAsi and mRNAsi), tumor mutational burden (TMB), and microsatellite instability (MSI) (Supplementary Fig. 5). Collectively, these findings suggest that high CD109 expression is associated with a tumor microenvironment enriched in macrophage and fibroblast-related features, along with molecular characteristics relevant to immune context and tumor progression.

### High CD109 expression is linked to resistance to immunotherapy and anticancer drugs

**Fig. 6:**
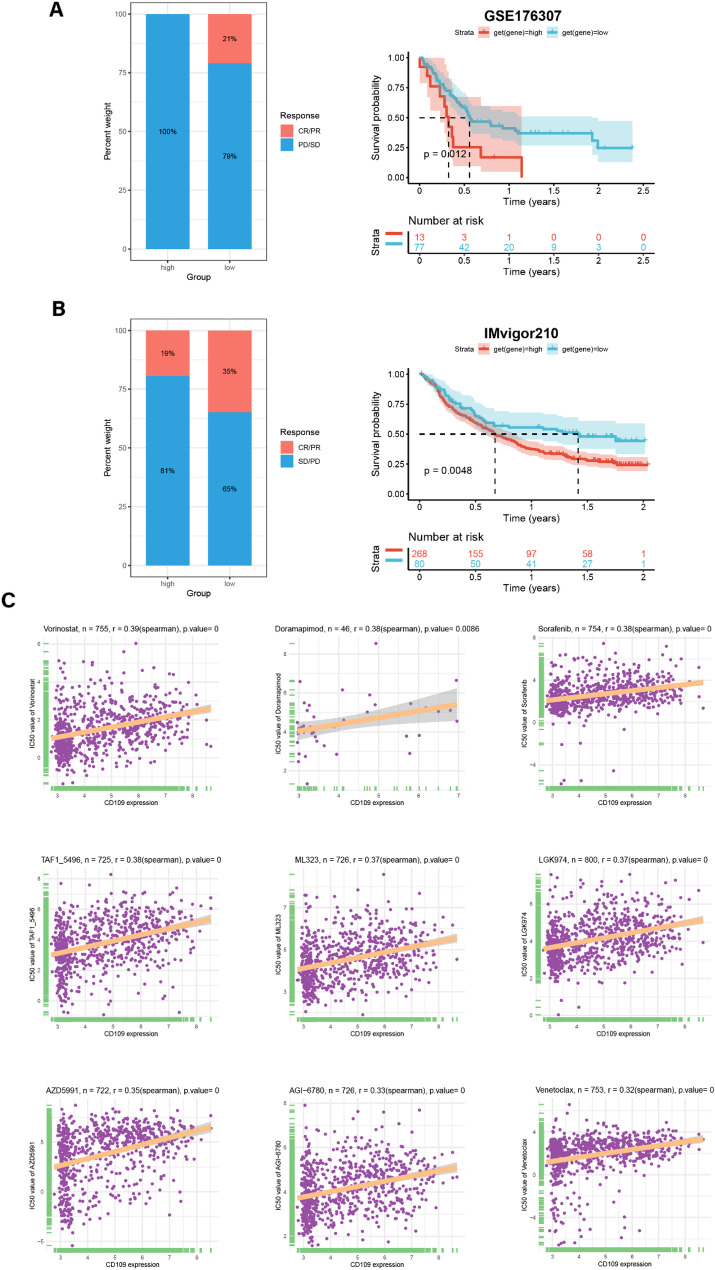
Drug sensitivity and immunotherapy response associated with CD109. **a** Relationship between CD109 and immunotherapy efficacy in independent GSE176307 and IMvigor210 cohorts, showing consistent trends of poorer response with high CD109 levels. **b** Scatterplot showing correlation between CD109 expression and the IC₅₀ values of representative anticancer drugs. *P  < 0.05, **P  < 0.01

Prompted by its link to an immunosuppressive microenvironment, we examined the association between CD109 expression and therapeutic efficacy. In the IMvigor210 (Urothelial Carcinoma) and GSE176307 (Metastatic Urothelial Cancer) immunotherapy cohorts, patients with high CD109 expression exhibited significantly poorer survival outcomes following PD‑L1 blockade (Fig. 6a–b). CD109 expression showed a pattern across response groups that was consistent with the survival analysis.

A comprehensive pan‑cancer drug‑response analysis using the GDSC database revealed that high CD109 expression was positively correlated with elevated IC50 values for 156 of 192 anticancer agents, suggesting reduced sensitivity to multiple anticancer compounds (Fig. 6c; Supplementary Table 4). This included diverse compounds such as Vorinostat (HDAC inhibitor), Doramapimod (p38 MAPK inhibitor), Sorafenib (multikinase inhibitor), and Venetoclax (BCL‑2 inhibitor). Collectively, these results suggest that high CD109 expression is associated with poorer immunotherapy outcome and reduced sensitivity to multiple classes of anticancer agents in an immunosuppressive context.

### Single‑cell characterization and in‑vitro functional assessment of CD109-associated macrophage polarization

**Fig. 7:**
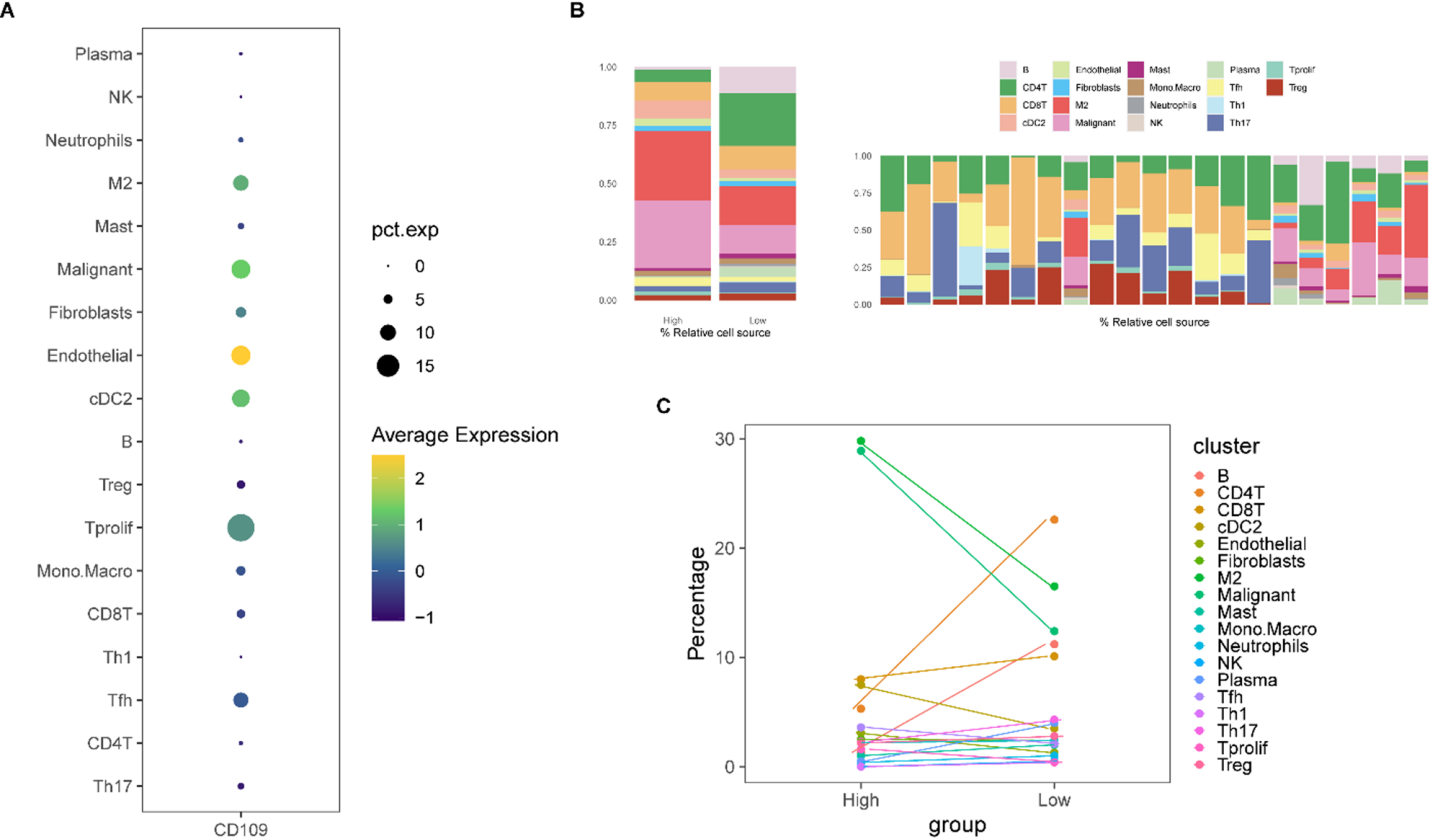
Single-cell transcriptomic characterization of CD109 expression. **a** UMAP visualization of CD109 distribution among malignant, endothelial, and macrophage populations. **b** Stacked bar graph depicting cell-type composition in CD109-high and CD109-low tumors. **c** Line chart comparing relative proportion differences

To characterize CD109 expression at single-cell resolution, we analyzed scRNA-seq data from two independent NSCLC cohorts (GSE99254 and GSE127465). Unsupervised clustering identified 18 cell types, which were annotated using COSG-identified marker genes. KEGG enrichment analysis showed that malignant cells exhibited elevated glycolytic activity, while M2 macrophages showed enrichment of IL-6 signaling pathways (Supplementary Fig. 6).CD109 was predominantly expressed in malignant cells, M2 macrophages, endothelial cells, and cDC2 cells (Fig. 7a). Samples were stratified into CD109-high and CD109-low groups based on overall expression. At the sample level, CD109-high samples showed altered cellular composition, including higher proportions of malignant cells, M2 macrophages, and fibroblasts, whereas CD109-low samples contained relatively more T-cell subsets (Fig. 7b). Quantitatively, fibroblasts and M2 macrophages were among the most enriched cell populations in CD109-high samples, whereas several T-cell subsets were relatively enriched in CD109-low samples (Fig. 7c). These differences should be interpreted cautiously, as the higher proportion of malignant cells in CD109-high samples may partly reflect variation in tumor cell content or tumor burden across samples, rather than a direct biological effect of CD109 itself. CellChat analysis revealed enhanced TGF-β-mediated signaling among malignant, endothelial, and macrophage clusters in CD109-high samples (Supplementary Fig. 7).

**Fig. 8:**
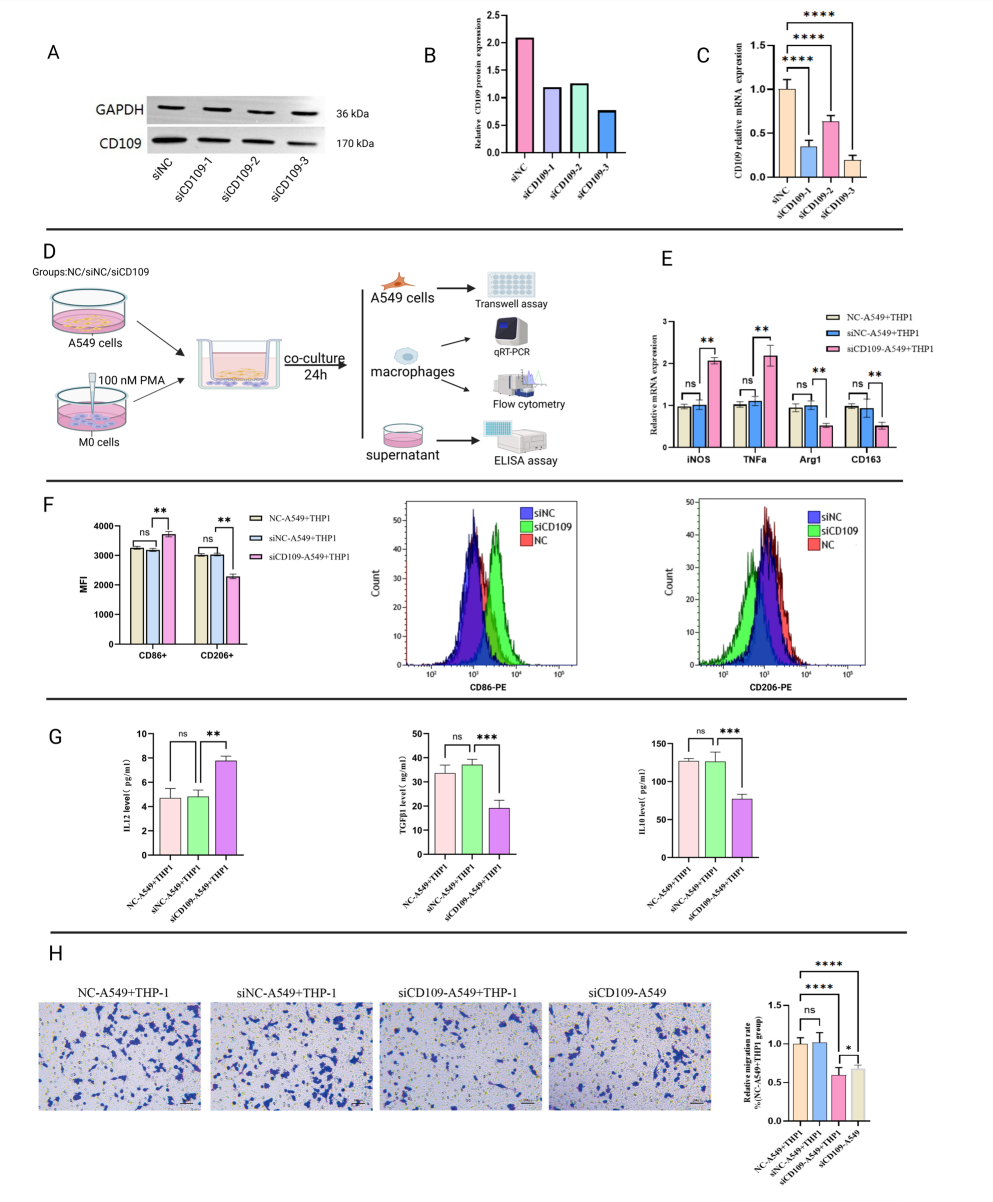
CD109 knockdown in A549 cells alters macrophage polarization and inhibits tumor cell migration. **a** Western blot of CD109 and GAPDH in A549 cells transfected with siNC or three CD109 siRNAs (siCD109‑1/‑2/‑3). **b** Densitometric quantification of CD109 protein normalized to GAPDH. **c** qPCR analysis of CD109 mRNA expression relative to siNC. **d** Schematic of the indirect co‑culture workflow. **e** qPCR analysis of macrophage polarization genes after 24 h of co‑culture showing increased expression of M1 markers (iNOS, TNF‑α) and decreased expression of M2 markers (Arg1,CD163) in the siCD109‑A549 + THP‑1 group compared with siNC‑A549 + THP‑1. **f** Flow cytometry analysis of macrophage surface markers after co‑culture, presented as mean fluorescence intensity (MFI) of CD86 and CD206 with representative histograms. **g** ELISA of co‑culture supernatants showing decreased levels of IL‑10 and TGF‑β1 and increased levels of IL‑12 in the siCD109‑A549 + THP‑1 group relative to siNC‑A549 + THP‑1. **h** Transwell migration assay showing representative crystal‑violet images and quantification of migrated A549 cells under co‑culture with THP‑1‑derived macrophages or monoculture conditions; scale bar, 50 μm. Data are presented as mean ± SD (*n* = 3). Statistical significance in panels e, f, and g was determined by one‑way ANOVA followed by Dunnett’s multiple‑comparison test versus the siNC‑A549 + THP‑1 group. Panel h was analyzed using two‑way ANOVA followed by Sidak’s post hoc test *P < 0.05, **P < 0.01, ***P < 0.001, ****P < 0.0001; ns, not significant

These single-cell findings are consistent with an association between CD109 expression and an immunosuppressive microenvironment in NSCLC, potentially involving both tumor-intrinsic expression and macrophage-related regulation. To test whether tumor cell derived CD109 directly influences macrophage polarization, we performed loss of function experiments in A549 lung adenocarcinoma cells, which exhibit high endogenous CD109 expression. CD109 was silenced in A549 lung adenocarcinoma cells using specific siRNAs. Efficient depletion was confirmed by western blot and qPCR. siCD109‑3 was applied for subsequent assays (Fig. 8a-c). In an indirect co‑culture system with THP‑1‑derived macrophages (Fig. 8d), CD109 knockdown promoted M1 polarization, as indicated by increased expression of iNOS and TNF-α and decreased expression of Arg1 and CD163 at the transcript level (Fig. 8e), increased surface expression of CD86 and reduced CD206 by flow cytometry (Fig. 8f), and a cytokine shift characterized by decreased IL‑10 and TGF‑β1 and elevated IL‑12 levels (Fig. 8g). Functionally, macrophages conditioned with siCD109‑A549 cells showed attenuated tumor cell migration promoting activity in transwell assays (Fig. 8h). To exclude the possibility that the reduced migration was merely caused by altered tumor cell growth, we further performed a CCK-8 assay over the same 24-h period as the Transwell experiment. CD109 knockdown did not significantly affect A549 cell viability at 24 h (Supplementary Fig. 8). Collectively, these data suggest that CD109 may promote M2‑type macrophage polarization and may be associated with a TGF‑β/IL‑10‑skewed cytokine profile, potentially contributing to an immunosuppressive TME.

## Discussion

The tumor immune microenvironment (TIME) constitutes a complex regulatory ecosystem where the equilibrium between immune surveillance and evasion is influenced by the dynamic interplay between malignant cells and stromal components [31–33]. In particular, the enrichment of M2-polarized TAMs acts as a critical barrier to effective immunotherapy, often precipitating primary or acquired resistance [34–36]. While immune checkpoint inhibitors targeting PD-1 or PD-L1 have transformed oncology, the limited response rates necessitate the identification of novel molecular features associated with this immunosuppressive landscape [37]. By integrating pan-cancer multi-omics profiling with in vitro functional validation, our study provides an integrative characterization of CD109, classically described as a TGF‑β co‑receptor, and supports an association between CD109 expression and immunosuppressive microenvironmental features across cancers, while functional evidence for macrophage-related effects was obtained in a lung cancer model.

Our pan-cancer analysis revealed CD109 upregulation across more than ten malignancies, including lung, liver, and glioma, expanding its known role beyond squamous and breast cancers [22, 27], Interestingly, the heterogeneous prognostic associations of CD109 across cancers likely reflect distinct immune landscapes. For example, CD109 may have different clinical associations in “inflamed” tumors compared with “immune-desert” or stromal-rich environments, although the underlying mechanisms remain to be determined. In addition, CD109’s correlation with TMB and MSI suggests that CD109 expression is associated with a tumor context characterized by genomic instability and altered immune surveillance. This observation aligns with recent evidence indicating that CD109 promotes tumor stemness and progression by stabilizing the gp130/IL-6Rα complex to activate the IL-6/STAT3 signaling axis [21, 22]. Furthermore, our enrichment analyses showed that CD109-high tumors were enriched in TGF-β, PI3K/AKT, and EMT pathways, supporting a possible role of CD109 as a molecular node associated with oncogenic signaling programs and microenvironmental remodeling. Taken together, these findings suggest that CD109 dysregulation is linked to both genomic and epigenetic alterations as well as distinct tumor-associated signaling states across cancers.

A key novelty of our study lies in characterizing the immunological dimension of CD109. Using scRNA-seq, we revealed that CD109 is not only expressed by malignant cells but also prominently upregulated in M2-like macrophages in NSCLC samples. This dual expression pattern suggests a potential interaction between tumor cell-intrinsic programs and myeloid‑associated interactions. Our CellChat analysis further suggested enhanced putative ligand-receptor communication, particularly along TGF‑β‑related signaling axes. This computational inference resonates with recent mechanistic studies demonstrating that tumor-secreted soluble CD109 (sCD109) can act via the FcγRI/SYK/NF-κB pathway to promote macrophage-mediated immunosuppression [30]. Accordingly, our bioinformatic and NSCLC single-cell data are consistent with the possibility that CD109 participates in macrophage-associated immunoregulatory interactions, although direct validation in additional tumor types and in vivo systems remains necessary.

Our functional experiments provided biological support for the bioinformatic associations observed in this study. Silencing CD109 in lung adenocarcinoma cells induced a moderate but consistent phenotypic shift in co‑cultured macrophages, characterized by a transition from a pro‑tumorigenic M2 state toward a more pro‑inflammatory M1‑like phenotype. This repolarization was evidenced by reduced expression of Arg1 and CD163, increased iNOS and TNF‑α levels, and a cytokine shift from an IL‑10/TGF‑β1‑dominant profile toward an IL‑12‑enriched state. Consistent with these immunologic changes, CD109 knockdown in A549 cells significantly decreased tumor cell migration and markedly weakened the migration‑promoting effect of THP‑1 co‑culture. These functional findings are in agreement with our pan‑cancer survival analyses and prior reports linking elevated CD109 expression to adverse clinical outcomes in specific tumor contexts [38, 39], although multivariable analyses indicated that such prognostic associations were retained only in selected cancers. Moreover, GSVA revealed enrichment of TGF‑β signaling and other malignant programs in CD109‑high tumors, which aligns with our co‑culture results showing reduced TGF‑β1 and IL‑10 secretion upon CD109 silencing. Collectively, these data suggest that CD109 may be involved in the establishment of a TGF‑β/IL‑10‑skewed, macrophage‑associated tumor promoting microenvironment, within the lung cancer experimental context examined here [40, 41].

Clinically, high CD109 expression was associated with blunted responses to PD-L1 blockade in urothelial cancer cohorts and correlated with broad-spectrum resistance to targeted therapies and chemotherapies, identifying a tumor subgroup characterized by reduced therapeutic responsiveness. This echoes preclinical findings in intrahepatic cholangiocarcinoma, where targeting CD109 reversed the immunosuppressive TIME and synergized with anti-PD-L1 therapy [30]. To enhance clinical utility, CD109 could be integrated into multiparametric prediction models alongside established biomarkers such as PD-L1 and TMB, potentially improving patient stratification. This perspective is supported by recent LUAD research showing that tumor-associated molecular signatures can reflect immune exclusion states and help predict immunotherapy efficacy [42]. Furthermore, simultaneous blockade of CD109 and conventional checkpoints may merit investigation as a promising strategy to modulate tumor-macrophage interactions, potentially shifting the microenvironment toward a less immunosuppressive state.

Our study has limitations that warrant further investigation. First, our in vitro validation was exclusively conducted using the A549 cell line. While A549 is a well-established model for NSCLC, future studies utilizing a broader panel of cell lines and in vivo models are warranted to further evaluate the generalizability of these findings. Second, our single-cell analysis was confined to NSCLC; expanding this resolution to other CD109-high malignancies would help determine whether these cellular interactions are generalizable beyond NSCLC. Finally, while CD109 acts as a negative regulator of TGF-β in keratinocytes [17, 43], our findings indicate a pro-tumorigenic role in lung cancer, which may be related to differential co-receptor availability or context-dependent signaling in the TME. Future studies utilizing anti-cleavage mutants or form-specific neutralizing antibodies are needed to dissect the specific contributions of the sCD109 ectodomain versus the membrane anchored protein.

In summary, our integrated analysis positions CD109 as a molecular feature associated with the immunosuppressive TIME. Our data indicate that CD109 expression is associated with M2-dominant macrophage features and with oncogenic pathway programs, including TGF-β- and STAT3-related signaling, with potential implications for tumor progression and therapy resistance. These insights warrant further investigation into CD109-targeted strategies, raising the possibility that modulating this macrophage-associated immune barrier may enhance the efficacy of current immunotherapies.

## Conclusion

Our study presents preliminary bioinformatic and experimental evidence indicating that CD109 expression is associated with immunosuppressive features of the TME across multiple cancer types. Functional validation in a lung cancer model further suggests that tumor cell-derived CD109 may influence macrophage polarization toward an M2‑like phenotype under in vitro co-culture conditions, with potential consequences for tumor cell migration and immunosuppressive features of the microenvironment. Taken together, these findings highlight the tumor type-specific prognostic relevance of CD109, with multivariable analyses further supporting its independent prognostic value only in selected cancers, and support further investigation of its potential role in macrophage-associated immune regulation and therapeutic responsiveness, while broader mechanistic generalization to other tumor types will require additional validation.

## Supplementary Information

Supplementary Material 1.

Supplementary Material 2.

## Data Availability

The datasets analyzed in this study are publicly available. Bulk RNA‑seq expression and corresponding clinical data were obtained from TCGA and GTEx project. Additional validation datasets were retrieved from the Gene Expression Omnibus. Single‑cell RNA‑seq datasets were downloaded from public repositories. All data generated during the *in vitro* experiments are included in this article and its supplementary information files. The analysis scripts are available from the corresponding authors upon reasonable request.

## Abbreviations

CAFs: cancer-associated fibroblasts
CCLE: Cancer Cell Line Encyclopedia
cDC2: conventional type 2 dendritic cell
DFI: disease-free interval
DSS: disease-specific survival
ECM: extracellular matrix
ELISA: enzyme-linked immunosorbent assay
EMT: epithelial-mesenchymal transition
ESTIMATE: Estimation of Stromal and Immune cells in Malignant Tumours using Expression data
FBS: fetal bovine serum
FDR: false discovery rate
GEO: Gene Expression Omnibus
GO: Gene Ontology
GPI: glycosylphosphatidylinositol
GSEA: gene set enrichment analysis
GSVA: gene set variation analysis
GTEx: Genotype-Tissue Expression
IC50: half-maximal inhibitory concentration
iTregs: induced regulatory T cells
KEGG: Kyoto Encyclopedia of Genes and Genomes
MFI: mean fluorescence intensity
MSI: microsatellite instability
mDNAsi: DNA methylation-based stemness index
mRNAsi: mRNA expression-based stemness index
NSCLC: non-small cell lung cancer
OS: overall survival
PCA: principal component analysis
PFI: progression-free interval
PI3K-Akt: phosphoinositide 3-kinase-protein kinase B
PMA: phorbol 12‑myristate 13‑acetate
qPCR: quantitative polymerase chain reaction
SDS-PAGE: sodium dodecyl sulfate‑polyacrylamide gel electrophoresis
scRNA-seq: single-cell RNA sequencing
siRNA: small interfering RNA
ssGSEA: single-sample gene set enrichment analysis
STAT3: Signal Transducer and Activator of Transcription 3
TAMs: tumor-associated macrophages
TCGA: The Cancer Genome Atlas
TGF-β: transforming growth factor-beta
TIME: tumor immune microenvironment
TISCH: Tumor Immune Single-cell Hub
TMB: tumor mutational burden
TME: tumor microenvironment
UMAP: Uniform Manifold Approximation and Projection
