# Supplementary material for: CD109 is associated with an immunosuppressive microenvironment and M2 macrophage polarization: pan-cancer analysis and functional validation
Source: BMC Cancer. 2026 May 1;26:760. doi: 10.1186/s12885-026-16100-4 (PMC13281367; doi:10.1186/s12885-026-16100-4)

**Supplementary Figure 1: Gene pairing expression.** CD109 expression in the TCGA cohort's paired tumor and nearby normal tissues.


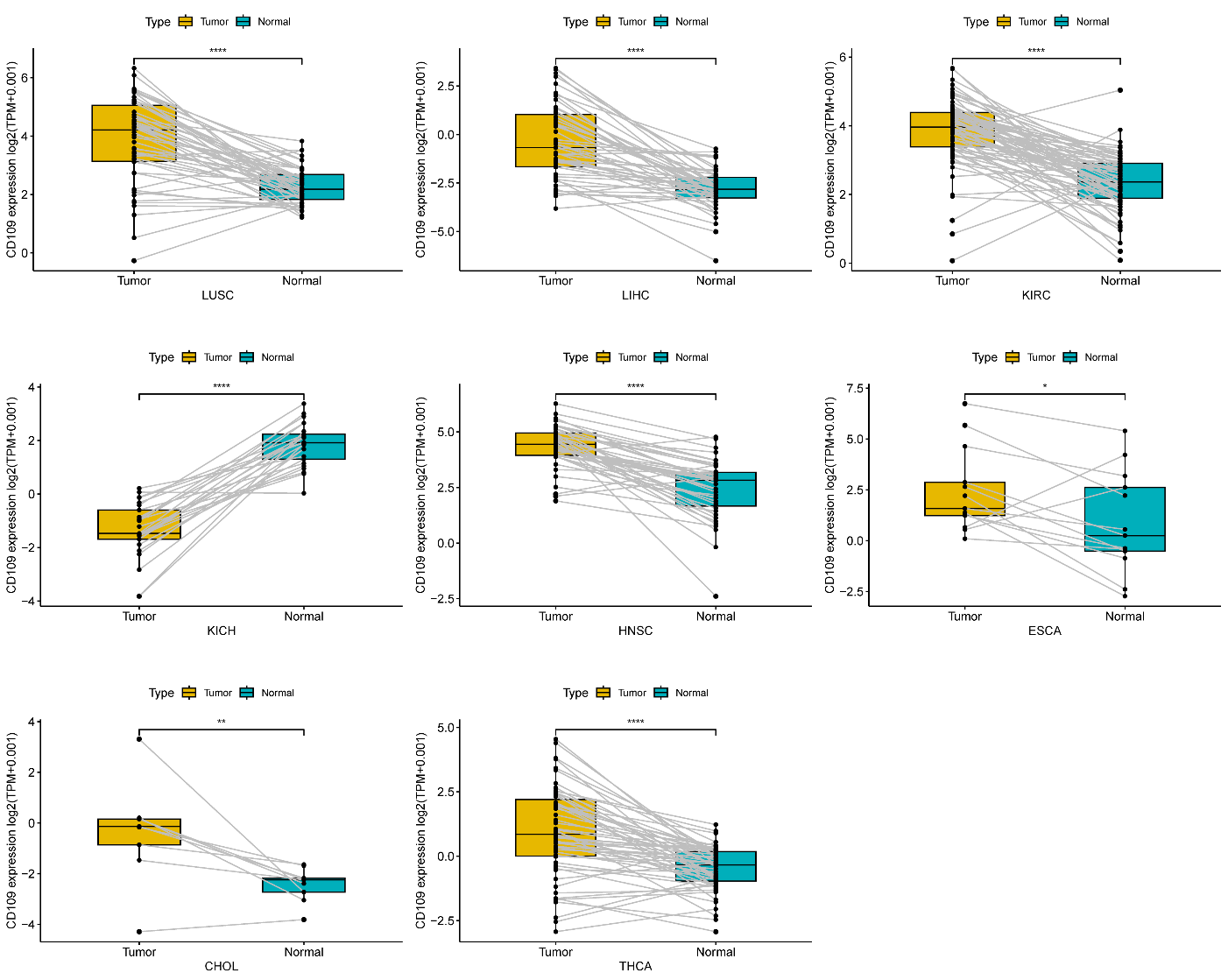


**Supplementary Figure 2: Extended Kaplan–Meier survival analyses of CD109 across cancers.** Kaplan-Meier curves for tumor types not shown in Fig. 2e–j.

**
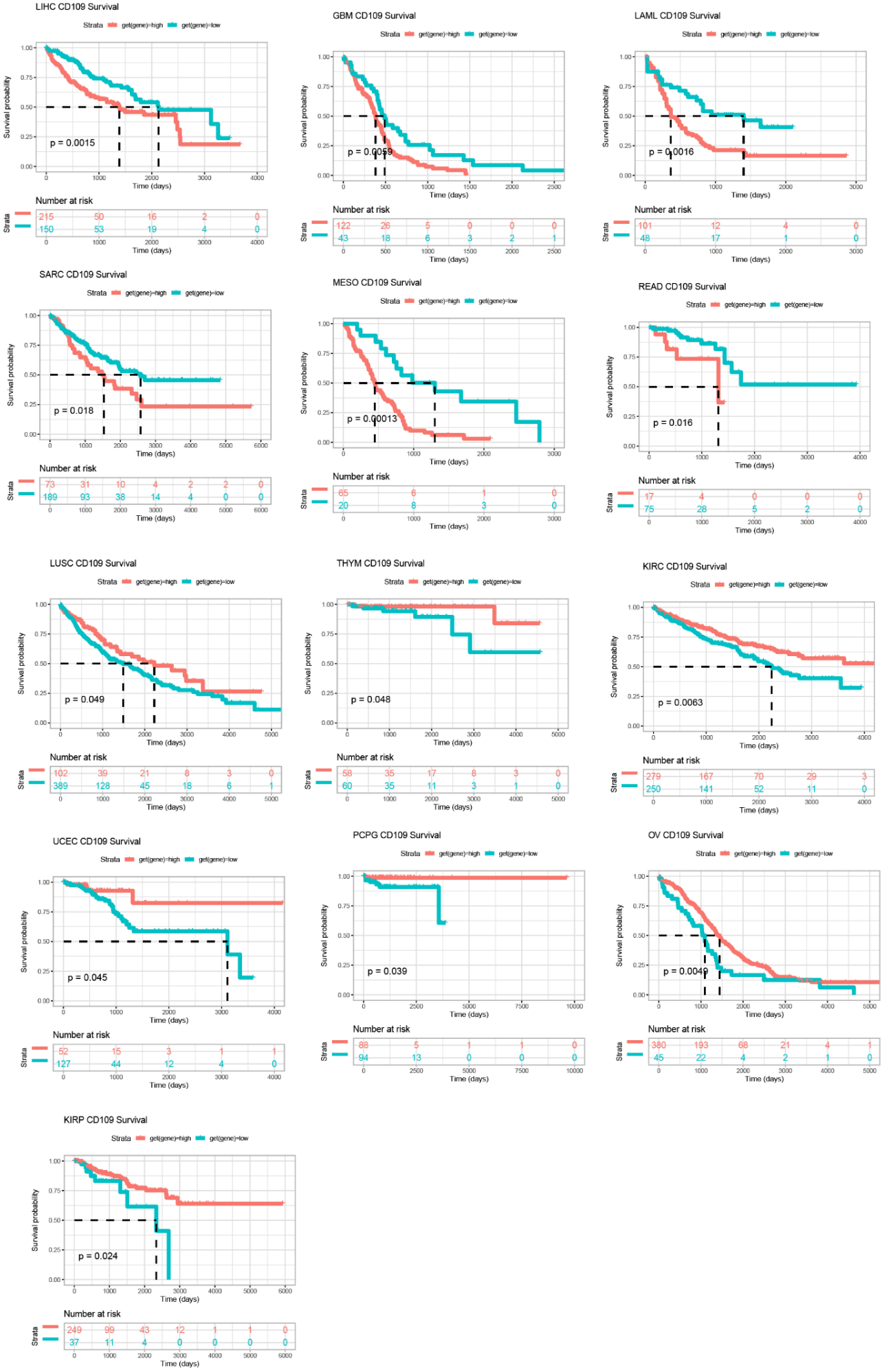
**

**Supplementary Figure 3: GSEA of CD109.** (a, b) The expression of the top 50 genes in LUAD is either positively or negatively correlated with CD109 expression. (c–e) The top 20 GSEA results of CD109 according to the LUAD GO (c), KEGG (d), and Reactome (e) pathways.


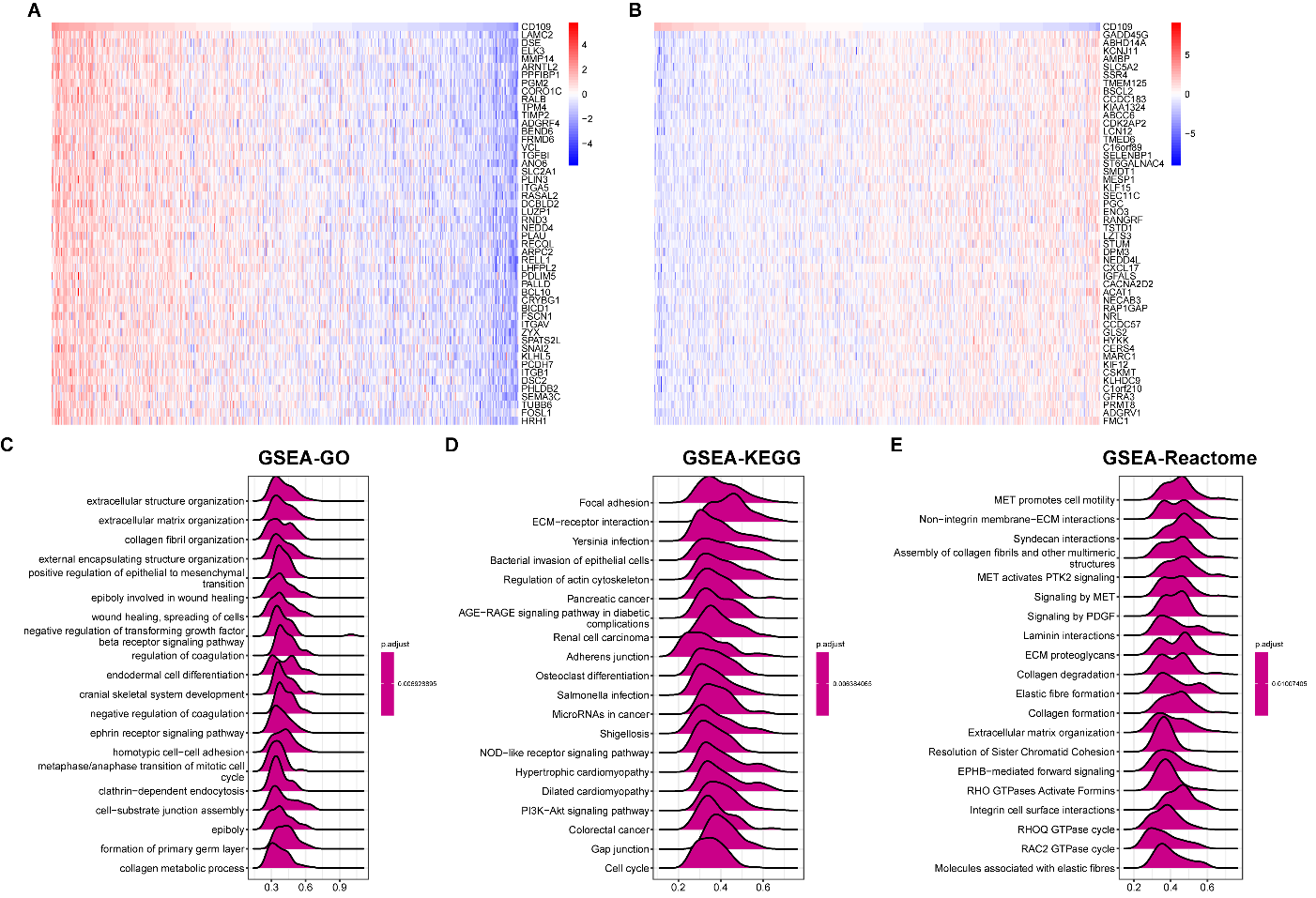


**Supplementary Figure 4: CD109 expression correlates with immunosuppressive genes.** (a) The relationship between CD109 expression and immunosuppressive genes in pan-cancer. (b, c) CD109 expression correlates with chemokines (b) and chemokine receptors. (c). *P < 0.05, **P < 0.01, ***P < 0.001, ****P < 0.0001.
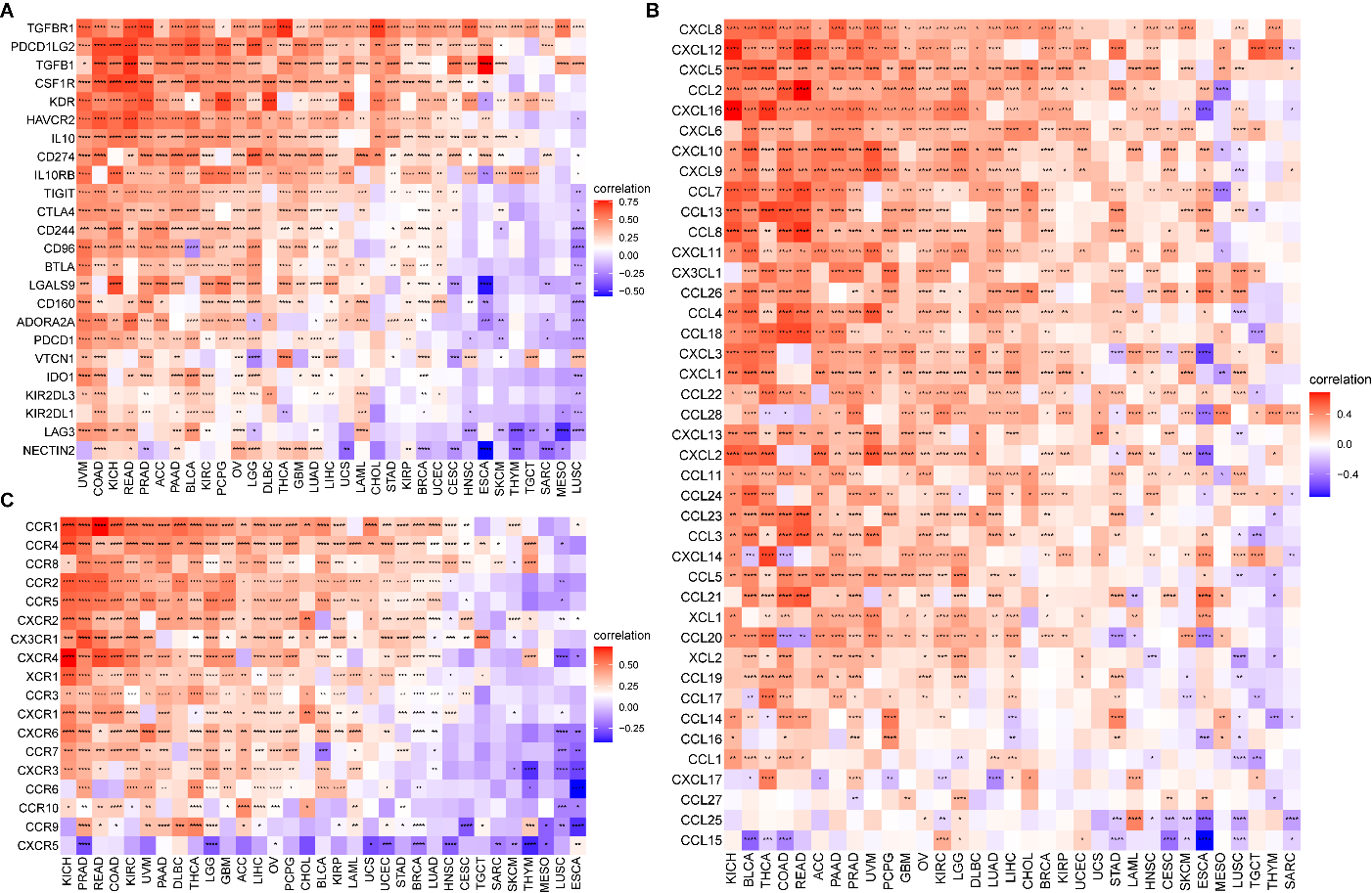


**Supplementary Figure 5: Correlation between CD109 expression and mDNAsi, mRNAsi, TMB, and MSI.** Correlation between CD109 expression and mDNAsi (a), mRNAsi (b), TMB (c), and MSI (d) in pan-cancer. *P < 0.05, **P < 0.01, ***P < 0.001, ****P < 0.0001.


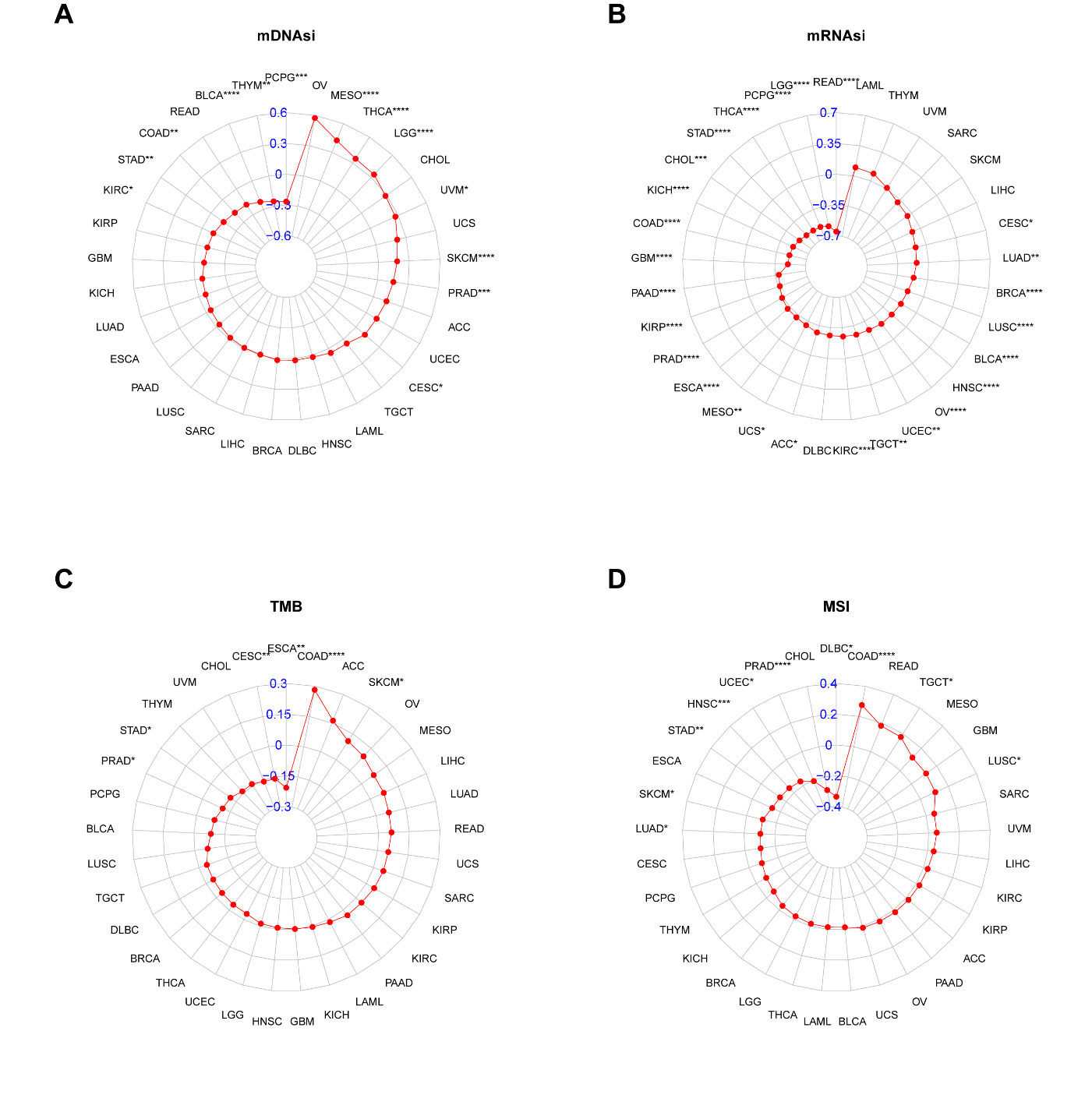


**Supplementary Figure 6: Basic single-cell analysis**. (a) UMAP visualization of single-cell transcriptomic data, colored according to cell type. (b) A volcano plot displaying each cell type's top 5 up- and down-regulated genes. The log2 fold change is shown on the y-axis, while the percentage difference is shown on the x-axis. (c) Heatmap showing each cell type's Hallmark pathway activity scores. Blue denotes low pathway activity, whereas red denotes strong pathway activity. (d) Heatmap showing how each cell type's top three marker genes are expressed. (e) Dotplot summarizing the results of KEGG in each cell types.


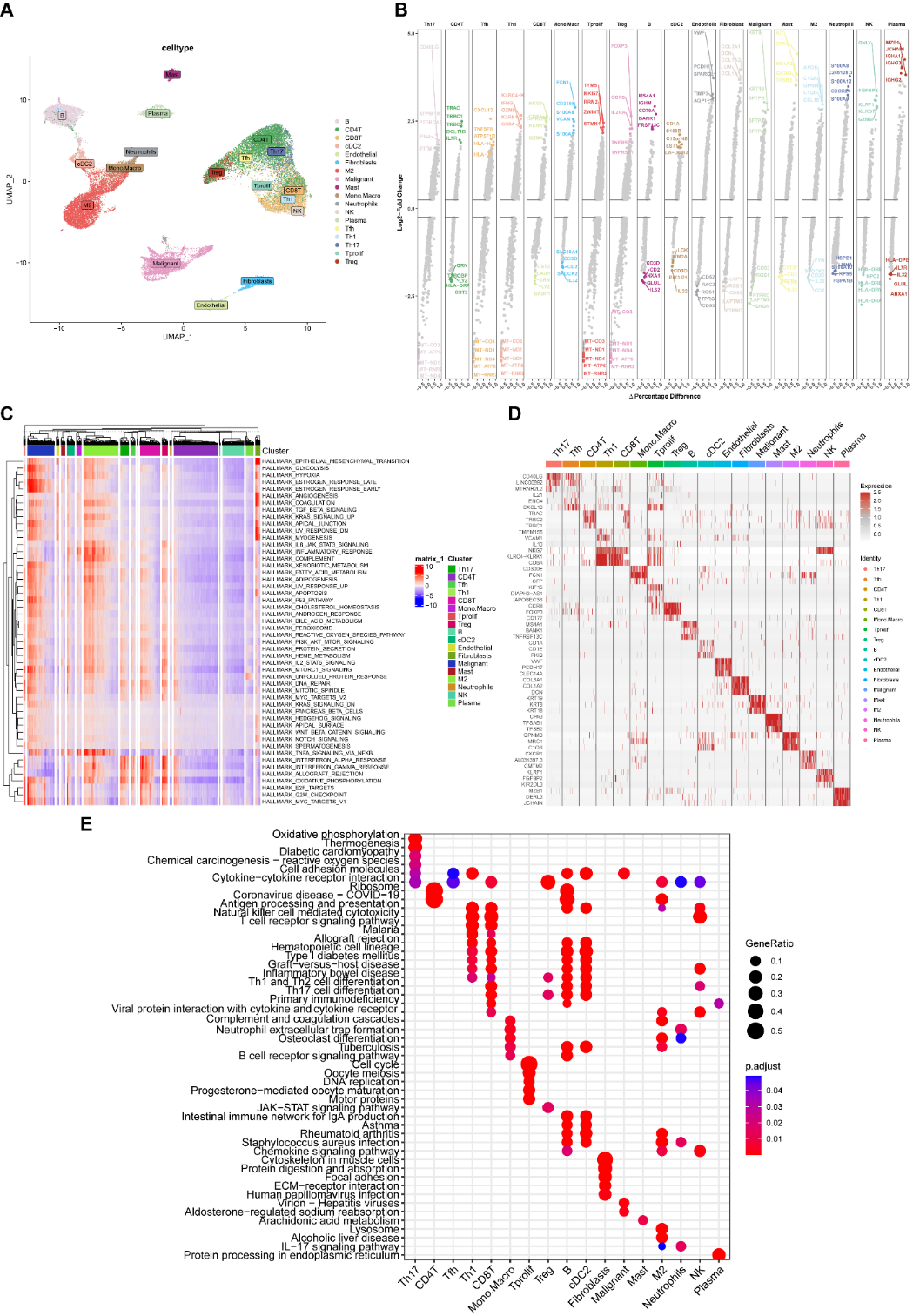


**Supplementary Figure 7: Analysis of cell-cell communication in high and low CD109 expression groups.** (a–b) Circos plots showing the number of contacts between various cell types in the groups with high and low CD109 expression (a and b, respectively). (c–d) Scatter plots showing the strength of incoming and outgoing interactions for each kind of cell in the groups with high (c) and low (d) CD109 expression. Total outgoing and incoming interaction strengths are shown by the X and Y axes, respectively. (e–f) Heatmaps depicting incoming and outgoing signaling patterns in the high (e) and low (f) CD109 expression groups. Each row is a ligand–receptor interaction, colored by its corresponding signaling weight. (g) Quantity of distinct interactions between various cell types in both CD109 high and low levels. (h) Violin plots illustrating the major signaling pathway genes' varying expression in high and low CD109 groups for various cell types. Expression is shown as red (blue) in the high (low) CD109 group.


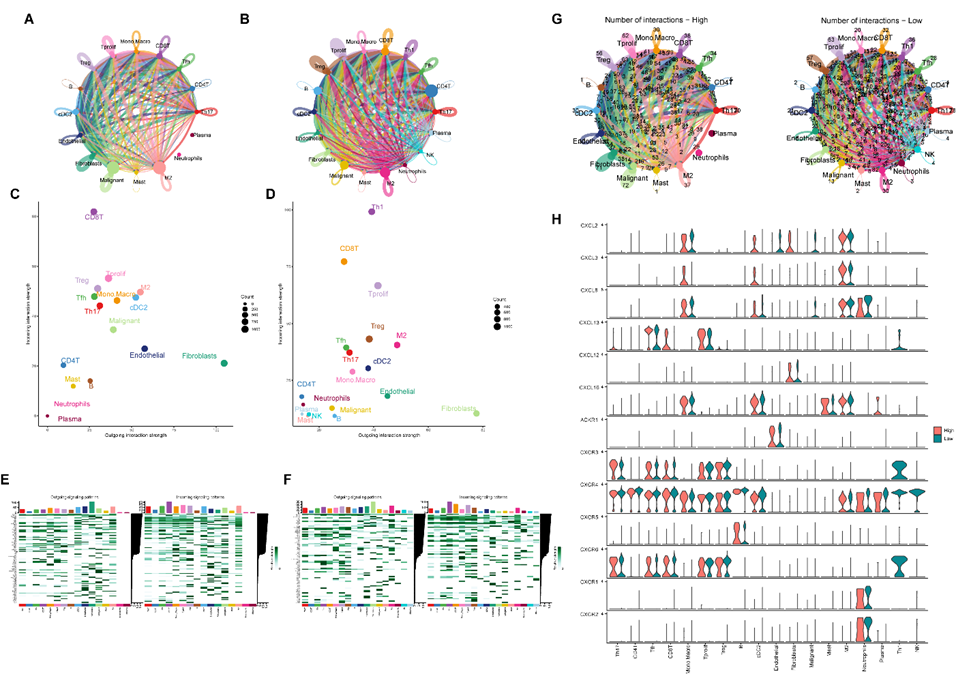


Supplementary Figure 8: CCK-8 assay of A549 cells after CD109 knockdown at 24 h


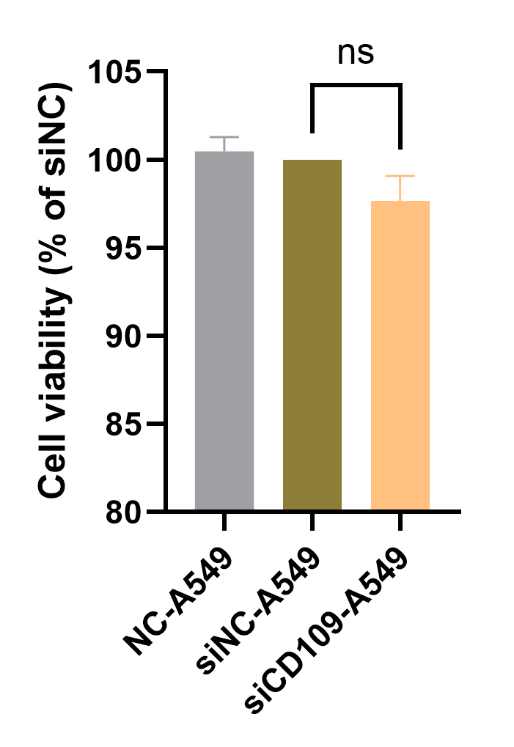

Supplement: Supplementary file 1 — Supplementary Material 1. [file 12885_2026_16100_MOESM1_ESM.docx]
