# Supplementary figures and images for: CD109 is associated with an immunosuppressive microenvironment and M2 macrophage polarization: pan-cancer analysis and functional validation
Source: BMC Cancer. 2026 May 1;26:760. doi: 10.1186/s12885-026-16100-4 (PMC13281367; doi:10.1186/s12885-026-16100-4)

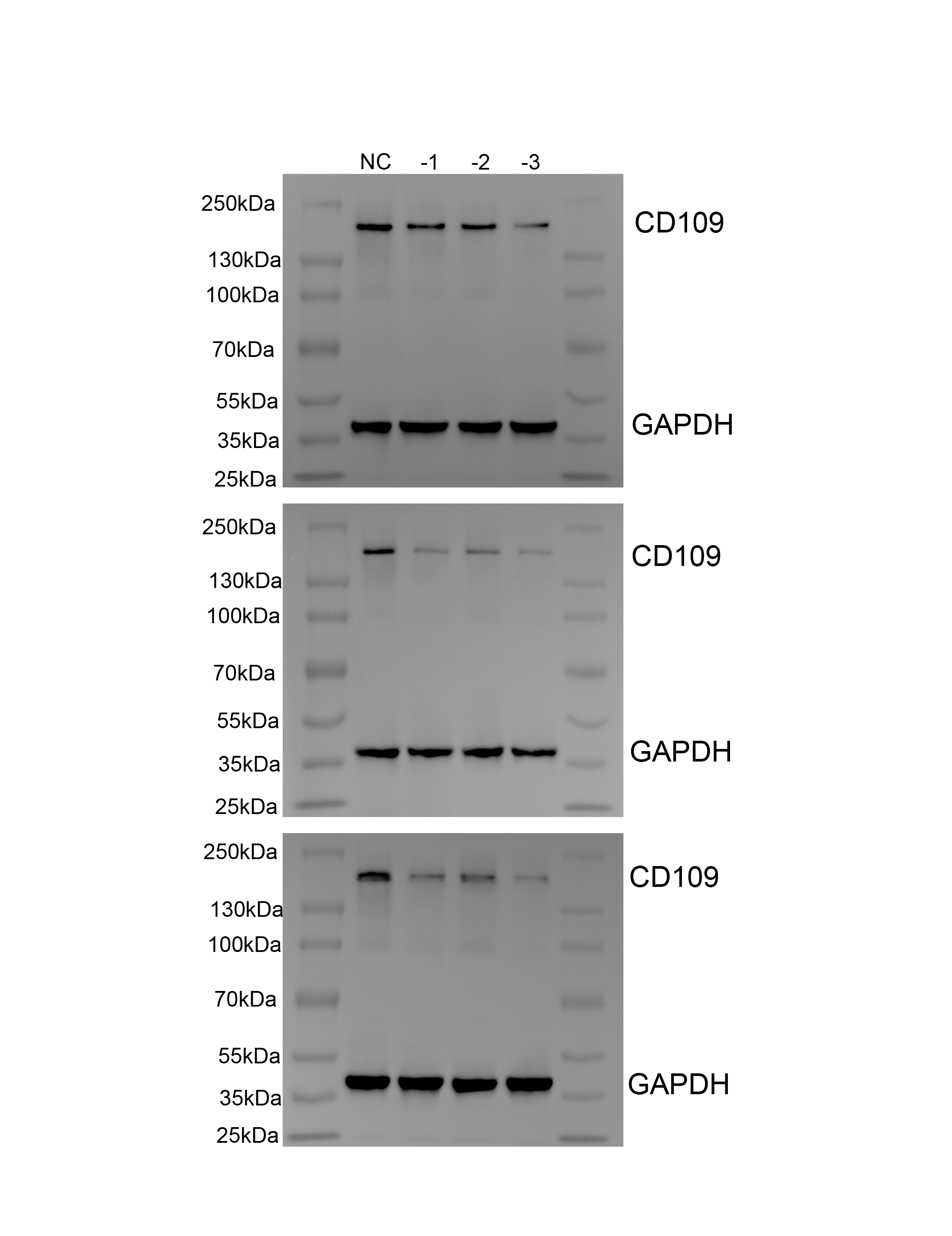

Supplement: Supplementary file 2 — Supplementary Material 2. [file 12885_2026_16100_MOESM2_ESM.tif]
